# Long-term cardio-metabolic effects of lifestyle intervention among obese Arab women: a 12-year follow-up of a randomized trial

**DOI:** 10.3389/fendo.2026.1786705

**Published:** 2026-03-24

**Authors:** Ofra Kalter-Leibovici, Nuha Younis-Zeidan, Havi Murad, Lizie Kimron, Angela Chetrit

**Affiliations:** 1 Gertner Institute of Epidemiology & Health Policy Research, Sheba Medical Center, Ramat-Gan, Israel; 2 Dina Recanati School of Medicine, Reichman University, Herzliya, Israel; 3 Sharon-Shomron Administration, Clalit Health Services, Hadera, Israel; 4 Biostatistics & Biomathematics Unit, Data & Analytics Division, Sheba Medical Center, Ramat-Gan, Israel; 5 Data & Analytics Division, Sheba Medical Center, Ramat-Gan, Israel

**Keywords:** cardio-metabolic risk factors, ethnicity, lifestyle intervention, long-term follow-up, obesity

## Abstract

**Background:**

Information on the long-term effects of lifestyle interventions after withdrawal among ethnic minority groups is sparse.

**Objective:**

To study the long-term cardio-metabolic effects of a 12-month intensive versus moderate culturally congruent lifestyle intervention after program termination.

**Methods:**

This is a long-term follow-up of a randomized controlled trial conducted between 2006 and 2008 in a primary care setting. Obese, non-diabetic Arab women aged 35–54 years were included (n=201). Both interventions promoted a calorie-restricted diet and regular physical exercise. The intensive intervention consisted of two monthly physical activity sessions and two monthly meetings with a dietitian. The moderate intervention, designed to follow usual care, consisted of five sessions/year with a dietitian. The main outcome measures were cardio-metabolic risk factors, measured during the trial and after its termination.

**Results:**

During a median follow-up of 12 years, women assigned to the intensive intervention had a more favorable BMI trajectory. They were also more likely to have a BMI <30.0 kg/m^2^ during follow-up, but this effect diminished over time; hazard ratios (95% confidence intervals) for the intensive vs. moderate intervention were 4.11 (2.17, 7.76) at the intervention end; 2.42 (1.47, 4.01) after 3 years, and 1.43 (0.81, 2.53) after 5 years. Among women with impaired fasting plasma glucose levels at baseline, the intensive intervention was associated with a more favorable glucose trajectory during follow-up. Nevertheless, the lifestyle intervention had no long-term effect on glucose levels among women with normal fasting glucose at baseline. No long-term effects were observed regarding blood pressure, plasma lipid levels, or the incidence of type 2 diabetes.

**Conclusion:**

A 12-month intensive lifestyle intervention among obese Arab women had a beneficial effect on BMI after intervention withdrawal, but the likelihood of having a BMI<30 kg/m^2^ diminished over time. It was also associated with an improvement in plasma glucose levels, observed only in women with impaired fasting glucose at baseline. The intervention did not produce sustained long-term improvements in other cardio-metabolic risk factors.

## Introduction

Some indigenous populations and ethnic minority groups, especially women, experience a higher prevalence of cardio-metabolic risk factors, including central obesity, elevated blood pressure, impaired glucose metabolism, and dyslipidemia (1–4).

Lifestyle intervention programs, which aim to achieve and maintain a normal body weight through a calorie-restricted diet and regular physical exercise, improve cardio-metabolic risk factor levels (5–9) and reduce the incidence of type 2 diabetes among at-risk individuals across diverse populations, in high- as well as low-and-middle income countries (9–13).

After the intervention ended, lifestyle intervention programs were associated with a reduced risk of type 2 diabetes among individuals with pre-diabetes, with a mean follow-up of 7 years. However, the effect size diminished over time (14). Nevertheless, there is limited information on the long-term effects of lifestyle intervention programs among indigenous and ethnic minority groups.

Women in Israel’s Arab minority have a higher prevalence of obesity, a greater risk of adult-onset diabetes, and present with diabetes at a younger age than women in the Jewish majority population (15, 16). We previously reported that a 12-month intensive, culturally congruent lifestyle intervention, which promoted a calorie-restricted diet and leisure-time physical activity, was more effective than a moderate intervention in increasing leisure-time physical activity, reducing body weight, fasting plasma glucose, and triglyceride levels among obese Arab women without diabetes who had one or more components of the metabolic syndrome (17). In this follow-up study, we aim to investigate the long-term cardio-metabolic effects of intensive versus moderate lifestyle interventions among these women after the program ends.

## Materials and methods

This is a follow-up study of a randomized trial with a parallel-group design, an active comparator, and 1:1 allocation. The study was conducted in a primary care setting of Clalit Health Services in two Arab communities in central Israel. Participants were 35–54 years old obese women (BMI between 30.0 and 40.0 kg/m^2^) without diabetes, who had one or more components of the metabolic syndrome (17). The study sample consisted of 201 Arab women recruited between 2006 and 2007. Trial Registration: ClinicalTrials.gov; Identifier NCT00273572.

After baseline evaluation, 100 women were randomly assigned to an intensive lifestyle intervention, and 101 to a moderate intervention. The intensive intervention comprised two monthly physical activity group meetings, one individual monthly session with a dietitian, and one monthly group meeting with a dietitian. The moderate intervention, designed to follow the usual care provided by the healthcare organization to individuals at risk, included two group meetings and three individual counseling sessions with a dietitian, all delivered within 12 months. Measurements of metabolic syndrome components, including body weight, waist circumference, blood pressure, fasting plasma glucose, triglycerides, and HDL cholesterol, were obtained at baseline and at 6 and 12 months (17).

Both interventions were culturally congruent and aimed to achieve at least 7% reduction in body weight by adhering to a calorie-restricted Mediterranean-style diet and engaging in regular leisure-time moderate physical activity for at least 150 minutes per week (17).

For the current follow-up study, the information collected during the trial was combined with information available in the electronic health records in Clalit Health Services to include data on body weight and blood pressure measurements, fasting plasma glucose, triglycerides, and HDL-cholesterol levels, diagnosis of hypertension, diabetes, or dyslipidemia, and chronic drug therapy, recorded after the trial completion. Information from Clalit Health Services was obtained for all participants from randomization until the data extraction date, transfer to another healthcare organization, or death. Any identifying information about the study participants was removed from the merged data file before analysis.

The primary outcome in the original study comprised the individual components of the metabolic syndrome, as defined by the National Cholesterol Education Program for women: fasting plasma glucose ≥110 mg/dL, fasting plasma triglycerides ≥150 mg/dL, fasting plasma HDL-C <50 mg/dL, blood pressure ≥130/≥85 mmHg, or waist circumference >88 cm. The metabolic syndrome was defined as the presence of three or more of these components (18). In the current study, the same components were the primary outcome, except for waist circumference. Because waist circumference is not routinely measured in primary care settings in Israel, we used body weight and calculated BMI to assess overweight and obesity during follow-up. Waist circumference and BMI are highly correlated and both associated with central adiposity (19). We also examined the trajectories of the individual metabolic component levels over time. Secondary outcomes included the incidence of pre-diabetes and diabetes. Pre-diabetes during follow-up was defined according to the American Diabetes Association (ADA) criteria for non-pregnant women (20). Presentation of diabetes during follow-up was defined as any two of the following: a diagnosis of diabetes documented in the patient’s health record, chronic hypoglycemic drug therapy, or blood test results meeting the ADA definition of diabetes (20). Because obesity was an inclusion criterion at the time of eligibility screening, we analyzed new events with BMI < 30 kg/m^2^ during follow-up.

### Statistical analysis

All analyses were performed using SAS (version 9.4, SAS Institute Inc., Cary, NC).

Comparisons of baseline characteristics between the intensive and moderate lifestyle intervention groups were made using the chi-square test for contingency tables, the independent-samples t-test for normally distributed continuous variables, or the Wilcoxon rank-sum test for non-normally distributed variables. Kaplan–Meier curves were constructed for each cardio-metabolic event, and differences between the intervention groups were assessed using the log-rank test.

Associations between the type of lifestyle intervention (intensive vs. moderate) and post-intervention cardio-metabolic trajectories were examined in linear mixed-effects models to account for repeated measures over time (21). The models were adjusted for baseline socio-demographic, anthropometric, and lifestyle characteristics. Pharmacotherapy was incorporated as a time-varying covariate within the linear mixed-effects models. Initiation and use of antihypertensive, lipid-lowering, and hypoglycemic medications were updated through follow-up to reflect changes in treatment status over time. This approach accounts for temporal changes in pharmacologic management that may influence cardio-metabolic trajectories and potentially attenuate between-group differences. Where appropriate, interaction terms between baseline cardio-metabolic parameters and intervention type were included to assess potential effect modification. Finally, multivariable Cox proportional hazards models were used to evaluate differences in time to cardiometabolic events, adjusting for baseline metabolic syndrome components and other relevant covariates. Independent variables associated with the outcome with a P value ≤ 0.2 in univariate analyses were included in the multivariable models. The proportional hazards assumption for the lifestyle intervention type was tested; when violated, interaction terms with follow-up time were included to account for time-dependent effects.

### Ethics considerations

This study was conducted in accordance with the principles of the Declaration of Helsinki. The Institutional Review Boards (IRBs) of Clalit Health Services and the Sheba Medical Center approved the study protocol. Both IRBs waived informed consent.

## Results

The baseline characteristics of the study participants were previously described (17). In brief, their mean age (SD) was 43.9 (5.7) years, their median years of education was 12 and their mean BMI was 33.9 (2.9) kg/m^2^. Fifty-one women (25.4%) had three or more of the metabolic syndrome components, seven women (3.5%) received statins, and 28 (13.9%) were receiving chronic drug therapy for hypertension. Only 12 women (6.0%) were engaged in leisure-time physical activity for at least 150 minutes/week before enrollment (**Table 1**).

**Table 1:** Baseline characteristics of the study participants.

	Total N=201	Lifestyle intervention intensity	
		Intensive N=100	Moderate N=101
Age, years, mean (SD)	43.9 (5.7)	43.8 (5.5)	44.0 (5.9)
BMI, kg/m^2^, mean (SD)	33.9 (2.9)	34.0 (3.1)	33.8 (2.8)
Leisure physical activity ≥150 min/week, N (%)	12 (6.0)	5 (5.0)	7 (6.9)
Metabolic syndrome* and its components:			
Waist circumference >88 cm, N (%)	198 (98.5)	98 (98.0)	100 (99.0)
Triglycerides ≥150 mg/dL, N (%)	46 (22.9)	26 (26.0)	20 (19.8)
FPG ≥110 mg/dL, N (%)	10 (5.0)	6 (6.0)	4 (4.0)
HDL-C <50 mg/dL, N (%)	104 (51.7)	47 (47.0)	57 (56.4)
BP ≥130/≥85 mmHg, N (%)	52 (25.9)	27 (27.0)	25 (24.8)
Metabolic syndrome, N (%)	51 (25.4)	26 (26.0)	25 (24.8)
Impaired glucose tolerance ^†^, N (%)	44 (21.9)	19 (19.0)	25 (24.8)
Any impaired glucose metabolism ^‡^, N (%)	50 (24.9)	24 (24.0)	26 (25.7)
Drug therapy, N (%)			
Lipid modifying	7 (3.5)	4 (4.0)	3 (3.0)
Antihypertensive	28 (14.0)	15 (15.0)	13 (12.9)

SD, standard deviation; BP, blood pressure; FPG, fasting plasma glucose; HDL-C, high-density lipoprotein cholesterol;.

* As defined by the National Cholesterol Education Program (18).

† Defined as plasma glucose ≥140 mg/dL and <200 mg/dL, 2 hours after a 75 g oral glucose load (18).

‡ Defined as either FPG ≥110 mg/dL or impaired glucose tolerance.

None of the comparisons between women assigned to the intensive versus moderate lifestyle interventions were statistically significant.

The median (interquartile range) follow-up period was 12.0 (11.5, 12.2) years among women assigned to the intensive lifestyle intervention, and 12.0 (11.6, 12.1) years among women assigned to the moderate intervention; p=0.27. Three women died during follow-up, of whom one was initially assigned to the intensive intervention, and two to the moderate intervention. An additional 13 women left the healthcare organization before the electronic health records data extraction date at Clalit Health Services (**Supplementary Figure 1**).

**Table 2** presents the incidence of metabolic syndrome and its components during follow-up among women with normal baseline measurements.

**Table 2:** Cardio-metabolic events during long-term follow-up^*^.

	Total N=201	Lifestyle intervention type		P ^†^
		Intensive N=100	Moderate N=101	
Metabolic syndrome and its components, N (%)				
BMI<30 kg/m^2^	73/186 (39.2)	40/91 (44.0)	33/95 (34.7)	0.062
Triglycerides ≥150 mg/dL	92/155 (59.4)	47/74 (63.5)	45/81 (55.6)	0.33
FPG ≥110 mg/dL	95/191 (49.7)	47/94 (50.0)	48/97 (49.5)	0.94
HDL-C <50 mg/dL	61/97 (62.9)	34/53 (64.2)	27/44 (61.4)	0.95
BP ≥130/≥85 mmHg	125/149 (83.9)	63/73 (86.3)	62/76 (81.6)	0.17
Metabolic syndrome	110/146 (75.3)	56/70 (80.0)	54/76 (71.1)	0.54
Any diabetes or pre-diabetes^‡^	107/151 (70.9)	56/76 (73.7)	51/75 (68.0)	0.32
Diabetes mellitus^§^	61/201 (30.3)	30/100 (30.0)	31/101 (30.7)	0.98
Initiation of chronic drug therapy, N (%):				
Hypoglycemic	55/201 (27.4)	27/100 (27.0)	38/101 (27.7)	0.15
Antihypertensive	79/173 (45.7)	39/85 (45.9)	40/88 (45.4)	0.59
Lipid modifying	109/194 (56.2)	52/96 (54.2)	57/98 (58.2)	0.57

* Defined among participants who did not have the parameter at baselineAbbreviations: BMI, body mass index; BP, blood pressure; FPG, fasting plasma glucose; HDL-C, high-density lipoprotein cholesterol;.

† Log-rank test.

‡ Following the American Diabetes Association (ADA) definition (20).

§ Defined as any two of the following: diagnosis of diabetes mellitus; chronic hypoglycemic drug therapy; or blood test results complying with the ADA definition of diabetes (20).

None of the comparisons between the lifestyle intervention types was statistically significant.

Sixty-one women (30.3%) presented with diabetes mellitus during follow-up; 30 women were initially assigned to the intensive lifestyle intervention and 31 to the moderate intervention (p=0.98).

Only 50% of women had at least one visit with a dietitian after program termination, and this proportion did not differ significantly across the lifestyle types.

**Figure 1:**
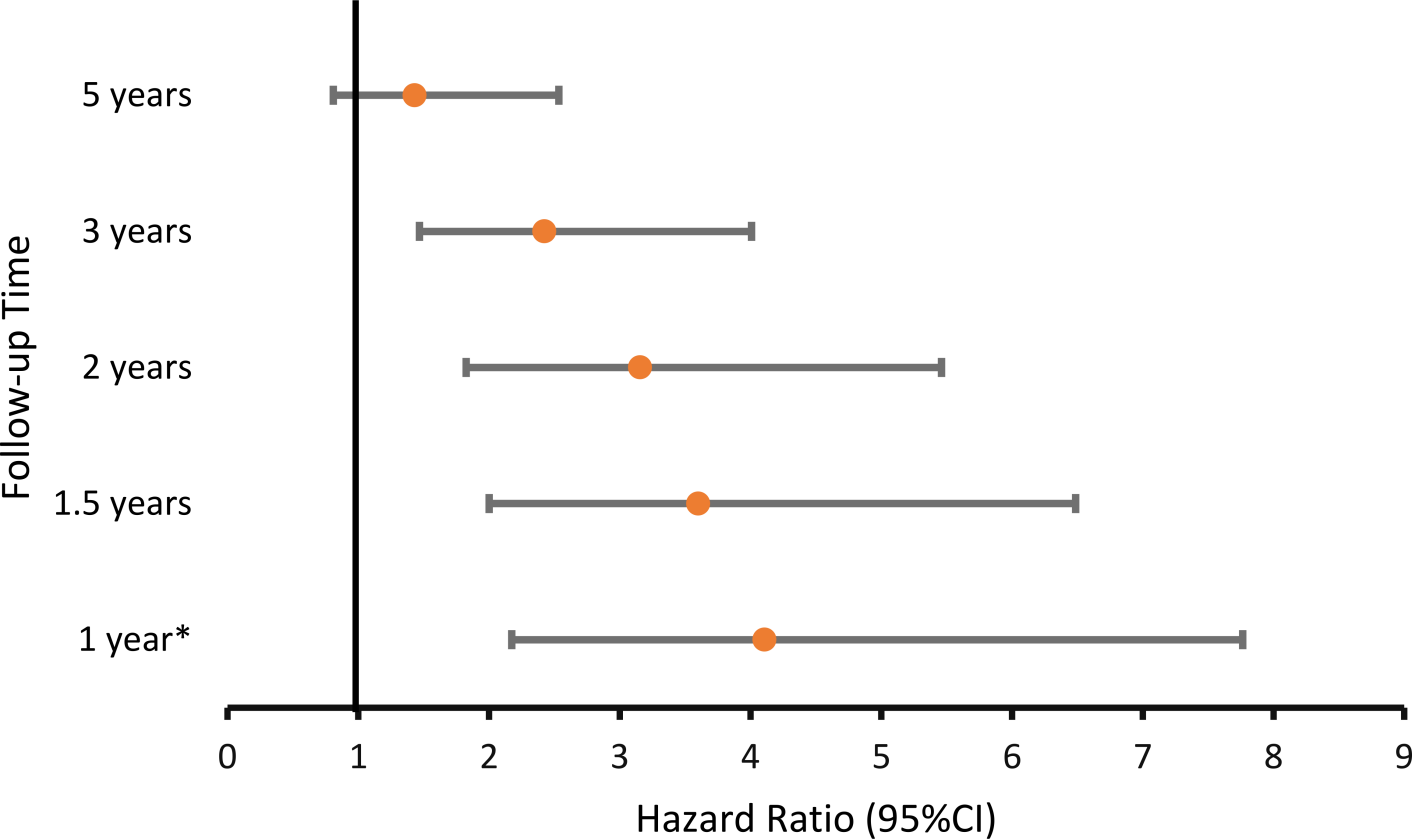
Hazard ratio for having BMI less than 30 kg/m^2^ at various time points during follow-up: Intensive versus moderate lifestyle intervention. *-At the end of the lifestyle intervention. CI, confidence interval.

To test the association between the type of lifestyle intervention and the hazard of having a BMI less than 30 kg/m^2^ during follow-up, a Cox proportional hazards model was fitted, adjusting for baseline age and BMI. In this model, the proportional hazards assumption was violated for the lifestyle intervention type and baseline BMI; therefore, interaction terms with follow-up time were included. Women assigned to the intensive lifestyle intervention were more likely to have a BMI <30 kg/m^2^ during follow-up than women assigned to the moderate intervention; however, this effect diminished over time and was no longer significant after 5 years since recruitment (**Table 3**, **Figure 1**).

**Table 3:** The effect of the lifestyle intervention type (intensive vs. moderate) and baseline BMI on the hazard of having a BMI<30 kg/m^2^ at various time points during follow-up *.

	Follow-up time, years						
	1 ^†^	1.5	2	3	5	7	9
	Hazard ratio (95% confidence interval)						
Lifestyle intervention type (Intensive vs. moderate)	4.11 (2.17, 7.76)	3.60 (2.00, 6.49)	3.16 (1.82, 5.46)	2.42 (1.47, 4.01)	1.43 (0.81, 2.53)	0.84 (0.38, 1.86)	0.50 (0.17, 1.46)
Baseline BMI, kg/m^2^ (1-unit increment)	0.48 (0.39, 0.58)	0.50 (0.42, 0.60)	0.52 (0.44, 0.62)	0.57 (0.49, 0.66)	0.69 (0.60, 0.79)	0.83 (0.71, 0.96)	1.14 (0.90, 1.44)

* Cox proportional hazards model adjusted for baseline age.

† End of the lifestyle intervention

A higher baseline BMI was associated with a lower hazard of achieving a BMI <30 kg/m^2^ over the first 7 years of follow-up (**Table 3**).

There were no significant associations between the type of lifestyle intervention and the risk of additional post-intervention cardio-metabolic events during follow-up (**Table 4**).

**Table 4:** The effect of the lifestyle intervention type on cardio-metabolic events during follow-up: Cox proportional hazards models.

	Plasma triglycerides ≥150 mg/dL	Blood pressure ≥130/85 mmHg	Plasma HDL-C <50 mg/dL	Pre-diabetes or diabetes*	Metabolic syndrome ^†^
Effect of the lifestyle intervention type (intensive vs. moderate), HR (95%CI)	1.18 (0.78, 1.79)	1.12 (0.77, 1.62)	1.05 (0.63, 1.75)	1.09 (0.73, 1.61)	0.90 (0.60, 1.35)
Model’s covariate adjustments	Baseline age, BMI, and triglycerides	Baseline age, SBP, years of education, interaction between SBP and follow-up time	Baseline age and HDL-C level	Baseline age, fasting plasma glucose, profession grading^‡^, and waist circumference.	Baseline age, marital status, abnormal baseline BP, triglyceride, and HDL-C, physical activity level at the end of the lifestyle intervention, interaction terms between marital status and baseline triglyceride level, and follow-up time
Number of observations included in the model	155	138	97	151	139

HR, hazard ratio; CI, confidence interval; BMI, body mass index; SBP, systolic blood pressure; HDL-C, high-density lipoprotein cholesterol;.

* Pre-diabetes during follow-up was defined according to the criteria of the American Diabetes Association (ADA) for non-pregnant women (20). Diabetes mellitus during follow-up was defined as any two of the following: diagnosis of diabetes in the patient’s health record, chronic hypoglycemic drug therapy, or blood test results compatible with the ADA definition of diabetes (20).

† As defined by the National Cholesterol Education Program (18).

‡ Profession grading based on a modified version of the International Classification of Occupations, ISCO-88, utilized by the Israel Central Bureau of Statistics. https://www.cbs.gov.il/en/publications/Pages/2015/Standard-Classification-of-Occupations-2011.aspx

We studied trajectories of repeated measurements of BMI, plasma glucose, triglycerides, HDL cholesterol, and blood pressure during follow-up, using linear mixed-effects models.

In a linear mixed-effects model adjusted for baseline age and BMI, and for baseline and end-of-intervention physical activity, we found that women assigned to the intensive lifestyle intervention had smaller increases in BMI over time than those assigned to the moderate intervention (**Table 5**).

**Table 5:** The effect of the lifestyle intervention type (intensive vs. moderate) on follow-up plasma lipids and blood pressure trajectories: Linear mixed-model analysis.

	Rate ratio (95% confidence interval)				
	BMI	Triglycerides	SBP	DBP	HDL-C
Lifestyle intervention type (intensive vs. moderate)	0.976 (0.961-0.991)	0.964 (0.901, 1.032)	0.994 (0.981, 1.007)	0.995 (0.981, 1.009)	1.014 (0.985, 1.045)
Adjustment for baseline and follow-up parameters	Baseline age, baseline BMI, baseline physical activity, and physical activity at the end of the lifestyle intervention	Baseline age, baseline physical activity, baseline triglycerides, and lipid modifying drug therapy during follow-up	Baseline age, baseline SBP, baseline waist circumference, baseline marital status, and hypotensive drug therapy during follow-up.	Baseline age, baseline DBP, baseline waist circumference, baseline marital status, and hypotensive drug therapy during follow-up.	Baseline age, baseline HDL-C, baseline cigarette smoking, and physical activity at the end of the lifestyle intervention.

SBP, systolic blood pressure; DBP, diastolic blood pressure; HDL-C, high-density lipoprotein cholesterol; FPG, fasting plasma glucose; RR, rate ratio; CI, confidence interval.

In another linear mixed-effects model adjusted for baseline age, waist circumference, marital status, and plasma glucose level measured 2 hours after an oral glucose load, as well as hypoglycemic drug therapy during follow-up, we found a significant interaction between the type of lifestyle intervention and the baseline fasting plasma glucose level. The intensive lifestyle intervention was associated with smaller increases in plasma glucose over time than the moderate intervention among women with baseline fasting plasma glucose levels ≥110 mg/dL, but not among those with lower baseline fasting plasma glucose levels (**Table 6**).

**Table 6:** The effect of the lifestyle intervention type (intensive vs. moderate) on follow-up plasma glucose trajectory by various baseline fasting plasma glucose levels: A linear mixed model analysis*.

Baseline fasting plasma glucose, mg/dL	Rate ratio (95% confidence interval)
87 (lowest quartile)	1.013 (0.990, 1.038)
100	0.979 (0.954, 1.005)
110	0.953 (0.911, 0.998)
120	0.929 (0.868, 0.994)

* Adjusted for baseline age, marital status, waist circumference, and plasma glucose level measured 2-hr post oral glucose load; and hypoglycemic drug therapy during follow-up.

There were no significant differences in the trajectories of triglycerides, HDL cholesterol, or systolic or diastolic blood pressure by intervention type (**Table 5**).

## Discussion

In this study, we described the long-term effects of a 12-month culturally congruent lifestyle intervention among obese Arab women without diabetes at baseline, after the program termination. We found that women assigned to the intensive intervention had a more favorable BMI trajectory. They were also more likely to be non-obese compared to those assigned to the moderate intervention, after program withdrawal. However, this effect did not last after five years. We also found that the glucose trajectory over time was more favorable among women assigned to the intensive lifestyle intervention than among those assigned to the moderate lifestyle intervention. However, this effect was observed only among women with impaired fasting plasma glucose levels at baseline. This finding is consistent with the results of the multivariable survival model, in which, among women with normal fasting glucose levels at baseline, the hazard of developing any impaired glucose metabolism during follow-up did not differ significantly by type of lifestyle intervention. By the end of follow-up, almost one-third of the participants presented with type 2 diabetes in both intervention groups. There were no significant differences between the two lifestyle interventions (intensive vs. moderate) in other cardio-metabolic risk factors, i.e., long-term blood pressure and plasma HDL cholesterol and triglyceride levels, after program withdrawal.

Our results differ from those reported in a meta-analysis of 19 randomized clinical trials, which found that lifestyle modification was associated with a 39% long-term relative risk reduction in diabetes (14). This discrepancy may reflect differences in intervention duration. While we tested a 12-month lifestyle intervention, three studies that achieved the most significant long-term reductions in diabetes risk had considerably longer interventions, up to 6 years (10–12). A systematic review of clinical trials testing lifestyle interventions with a physical activity component among obese individuals found that long-term interventions induced greater weight loss than short- or intermediate-term interventions (5). After weight loss, lifestyle interventions that combine diet and physical exercise components also show significant benefits for weight maintenance (22). Adopting and maintaining healthy lifestyle habits may pose a greater challenge among ethnic minority groups. A study among Kuwaiti individuals with cardio-metabolic risk factors reported that about two-thirds failed to adhere to a healthy diet and physical activity regimen due to personal (e.g., lack of motivation or time) and cultural barriers (e.g., social gatherings and high-fat traditional cuisine) (23). Thus, the reported benefits of medical nutritional therapy provided by a dietitian for weight loss and improvements in blood pressure, fasting blood glucose, and lipid levels (24) may be more pronounced among ethnic minority groups facing these barriers.

Even in the era of widespread pharmacotherapy prescribed for people with obesity, professional organizations recommend nutritional advice and regular exercise to achieve sustained control of adiposity and avoid drug-related side effects (25, 26). In Israel, individuals with cardio-metabolic risk factors are entitled to long-term nutritional advice under the National Health Insurance Law. The fact that only half of the participants had at least one visit to a dietitian after the intervention ended highlights the need for taking an outreach approach. As the rate of Arab women engaged in any leisure physical activity in Israel is very low (27), adoption of national policies that enable Arab women to engage in physical exercise is crucial for effective and sustainable cardio-metabolic risk prevention. In a 10-year follow-up of the Diabetes Prevention Program in the US, lifestyle modification added more quality-adjusted life-years than metformin (28). Such evidence should motivate healthcare organizations to invest more effort in providing long-term, culturally congruent, and proactive lifestyle modification support to achieve an effective cardio-metabolic risk modification among at-risk ethnic minority groups.

Our study has some limitations, including a modest sample size, designed to test the effectiveness of the 12-month intervention. This sample may be underpowered to test smaller, long-term cardio-metabolic effects. In addition, other undocumented interventions that might have happened after the end of the 12-month intervention could have an effect on the long-term cardio-metabolic outcomes. We did not have follow-up information on dietary advice provided outside the healthcare system, and information on regular physical exercise is not routinely documented in patient health records.

Nevertheless, this study provides significant information on the long-term effects of a 12-month intensive lifestyle modification among at-risk individuals from the Arab ethnic minority in Israel.

Our results show that a 12-month intensive lifestyle intervention achieves limited long-term improvements in cardio-metabolic risk among obese Arab women. Given the high burden of obesity and cardio-metabolic morbidity, there is a strong need to design and test interventions aimed at achieving a sustainable reduction of cardio-metabolic risk among these women.

## Data Availability

The datasets presented in this article are not readily available because restrictions apply to the availability of all data generated or analyzed during this study to preserve patient confidentiality. The corresponding author will, on request, detail the restrictions and any conditions under which access to some data may be provided. Requests to access the datasets should be directed to ofral@gertner.health.gov.il.
