# Supplementary material for: Long-term cardio-metabolic effects of lifestyle intervention among obese Arab women: a 12-year follow-up of a randomized trial
Source: Front Endocrinol (Lausanne). 2026 Mar 24;17:1786705. doi: 10.3389/fendo.2026.1786705 (PMC13053252; doi:10.3389/fendo.2026.1786705)
Supplement: Supplementary file 1 [file DataSheet1.docx]

Assessed for eligibility: (n=410)

1^st^ step screening only (n=180)

1^st^ and 2^nd^ step screening (n=230)

Excluded (n=209)

Not meeting inclusion criteria (n=128)

Refused to participate (n=81)

After 1^st^ step screening (n=76)

After 2^nd^ step screening (n=5)

## Enrollment

Analyzed (n=101)

***Long-term Follow- Up Analysis***

Analyzed (n=100)

## Post Intervention Follow-Up

Discontinued follow-up before Clalit data extraction date: (N=5)

Because of death: (n=2)

Because of transferred to

another healthcare organization:

(n=3)

Discontinued follow-up before Clalit data extraction date: (N=11)

Because of death: (n=1)

Because of transferred to another

healthcare organization: (n=10)

Analyzed (n=101)

## End of Intervention Analysis

Analyzed (n=100)

Lost to follow-up (n=0)

Discontinued intervention: (n=14)

Because of personal reasons

(n=9)

Because of lack of motivation

(n=5)

Lost to follow-up (n=1)

Failed contact

Discontinued intervention because of lack of motivation

(n=6)

Allocated to intensive lifestyle intervention: (n=100)

Received allocated intervention

(n=97)

Did not receive allocated intervention because of personal reasons

(n=3)

Allocated to moderate lifestyle intervention: (n=101)

Received allocated intervention

(n=98)

Did not receive allocated intervention because of disappointment with the assigned intervention

(n=3)

## Allocation

## Follow-Up

Randomization
